# A Case of Eosinophilic Pustular Folliculitis since Birth

**DOI:** 10.3390/children8010030

**Published:** 2021-01-07

**Authors:** Satoshi Yoshida, Kazuki Yatsuzuka, Kenji Chigyo, Yuta Kuroo, Koji Takemoto, Koji Sayama

**Affiliations:** 1 Department of Dermatology, Ehime University Graduate School of Medicine, Ehime 791-0295, Japan; ehimeehime101@gmail.com (S.Y.); y.kuroo@gmail.com (Y.K.); sayama@m.ehime-u.ac.jp (K.S.); 2 Department of Pediatrics, Ehime Prefectural Niihama Hospital, Ehime 792-0042, Japan; kenji1991911@gmail.com (K.C.); takemoto.koji.1970@gmail.com (K.T.)

**Keywords:** eosinophilic pustular folliculitis, transient neonatal pustular melanosis, erythema toxicum neonatorum, neonatal eosinophilic pustulosis

## Abstract

A newborn male infant presented with multiple pustules and erosions with erythema involving his scalp and forehead at birth. One week after birth, new pustules continued to appear, forming crusted, ring-shaped plaques with pigmentation. Tests for possible pathogens were negative. Tzanck smear and skin biopsy revealed pustules beneath the stratum corneum at sites corresponding to hair follicles, which contained eosinophils and neutrophils. Taken together, a diagnosis of eosinophilic pustular folliculitis (EPF) was made. The pustules on the head disappeared rapidly with topical corticosteroid treatment, although new eruptions were still observed on the trunk about one month after birth. To our knowledge, only two cases of EPF since birth have been reported to date. Here, we also discuss the differential diagnosis of noninfectious pustular diseases at birth, including erythema toxicum neonatorum and transient neonatal pustular melanosis. These diseases, and EPF, may present with very similar clinical symptoms at birth, and the Tzanck test or biopsy may be required for differential diagnosis.

## 1. Introduction

Pustules are rarely present at birth. Regardless of the time of presentation, pustular diseases can be infectious or noninfectious. Infectious pustular diseases may be life-threatening and require a particularly early diagnosis. These diseases can be differentiated through the identification of bacteria, fungi, and viruses [1].

Noninfectious pustular diseases include erythema toxicum neonatorum (ETN), transient neonatal pustular melanosis (TNPM), and eosinophilic pustular folliculitis (EPF). ETN and TNPM usually resolve spontaneously, whereas EPF often requires treatment. These diseases may present with very similar clinical symptoms at birth and must be differentiated for effective, targeted treatment. Here, we describe a rare case of EPF since birth, along with differential diagnosis from other diseases associated with pustules seen at birth.

## 2. Case Presentation

A male baby was born with numerous pustules on his scalp and forehead. His 35-year-old mother had a history of Graves’ disease and Moyamoya disease, which were treated with propylthiouracil and aspirin, and he was born by cesarean section at term. The perinatal period was uneventful, and the infant had a birth weight of 2700 g and Apgar scores of eight and nine at 1 and 5 min, respectively. 

**Figure 1:**
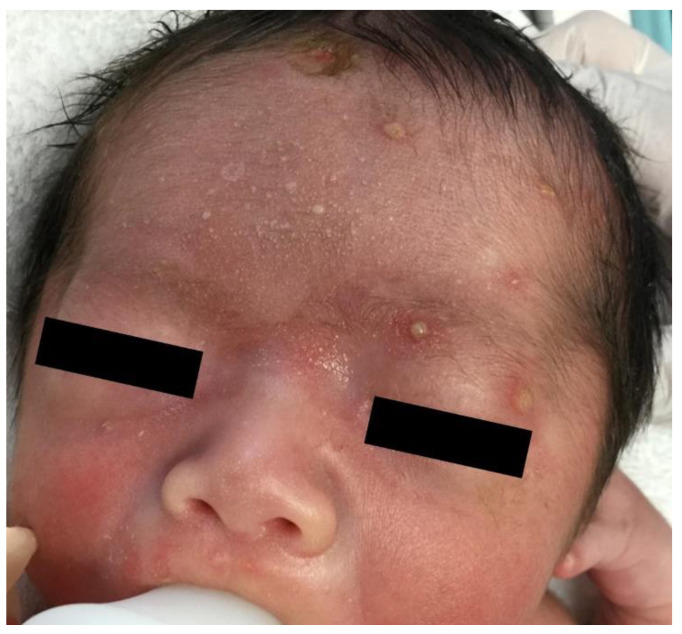
At birth, the patient developed multiple pustules measuring 1–5 mm and a few erosions with erythema on his scalp and forehead. Some pustules were crusted.

At birth, multiple pustules measuring 1–5 mm and a few erosions with erythema were observed on his scalp and forehead (Figure 1). There were no systemic symptoms, such as fever or dyspnea. His blood showed a normal C-reactive protein level (0.01 mg/dL; reference range: 0–0.4 mg/dL), elevated white blood cell count (12,500/μL; reference range: 3400–9400/μL), normal eosinophil count (500/μL; reference range: 0–658/μL), normal β-D-glucan level (9.9 pg/mL; reference range: <20.0 pg/mL), elevated thyroid-stimulating hormone level (10.76 μIU/mL; reference range: 0.34–3.88 μIU/mL), normal free triiodothyronine level (1.05 ng/dL; reference range: 0.95–1.74 ng/dL), and decreased free thyroxine level (1.82 pg/mL; reference range: 2.13–4.07 pg/mL). The Tzanck test results of the pustules showed no multinucleated giant cells. Bacterial and fungal cultures of the pustules were performed, and treatment was started as infectious pustulosis before the results were obtained. The infant was treated with topical nadifloxacin and ketoconazole, and infusion of acyclovir, cefazolin sodium, and vancomycin hydrochloride. 

**Figure 2:**
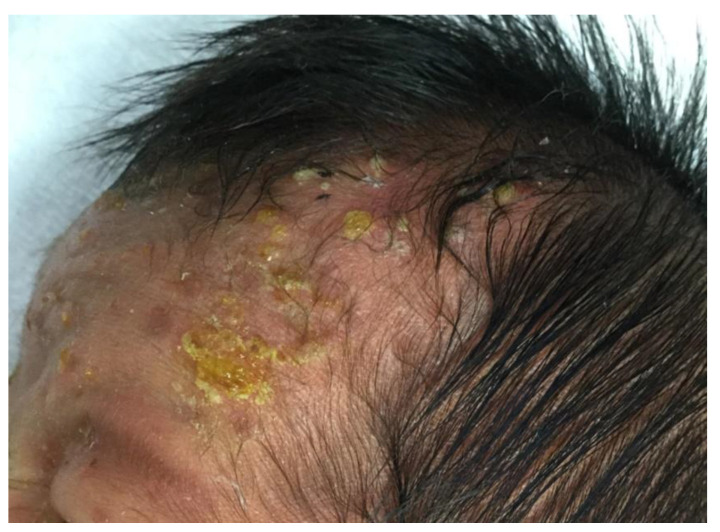
One week after birth, the patient developed ring-shaped plaques consisting of pustules, crusts, and erythema on his scalp and forehead. Pigmentation was seen on the forehead, consistent with previous erythema and pustules.

**Figure 3:**
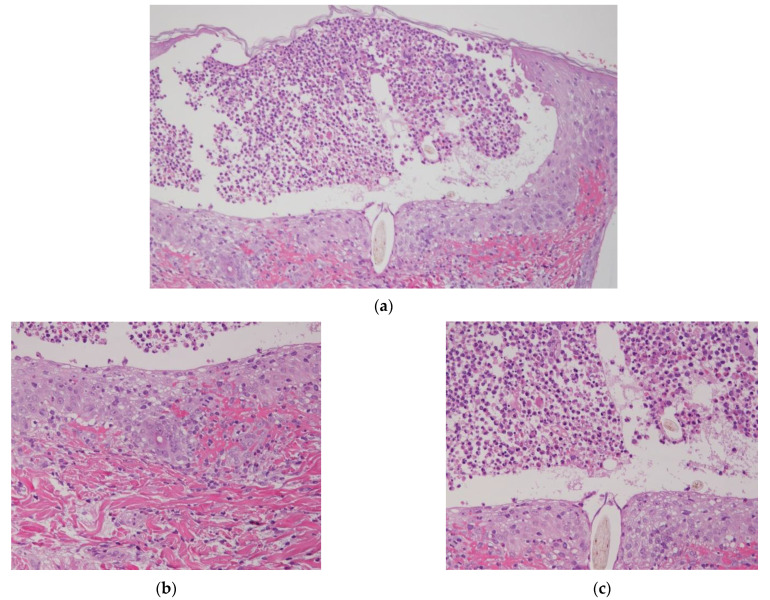
Hematoxylin and eosin staining. (**a**) A skin biopsy revealed pustules beneath the stratum corneum, which coincided with the epidermal opening of hair follicles. Original magnification ×100. (**b**) A small amount of inflammatory cell infiltration, such as eosinophils and lymphocytes, was also seen around the hair follicles in the dermis, and between the dermal collagen fibers. Original magnification ×200. (**c**) The pustules contained eosinophils, neutrophils, and hairs, suggesting the destruction of the hair follicles. Original magnification ×200.

One week after birth, new pustules continued to appear, forming crusted, ring-shaped plaques with pigmentation (Figure 2). Bacterial and fungal cultures were found to be negative, and the infusion of antibiotics and antivirals was discontinued. To make an accurate diagnosis, we additionally performed a Tzanck smear of the pustule and a skin biopsy. The Tzanck smear showed a large number of eosinophils and neutrophils, and the skin biopsy revealed pustules beneath the stratum corneum at sites corresponding to hair follicles, which contained eosinophils and neutrophils (Figure 3a,c). A small amount of eosinophilic infiltration was also detected around hair follicles in the dermis, and between the dermal collagen fibers (Figure 3b). 

**Figure 4:**
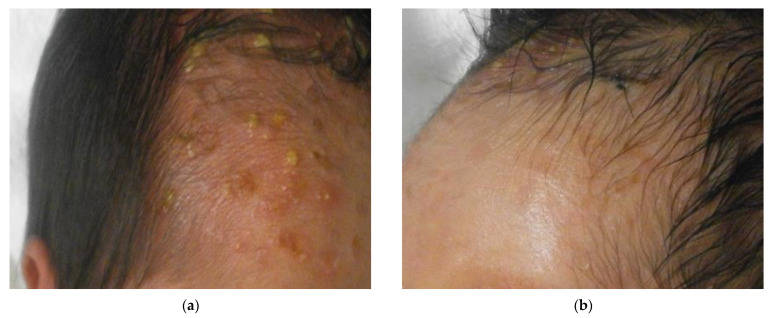
(**a**) New pustules continued to appear on the right forehead after topical ketoconazole treatment. (**b**) The pustules and erythema disappeared on the left forehead after topical corticosteroid treatment.

Finally, we made a diagnosis of EPF based on a combination of clinical symptoms, Tzanck smear, and skin biopsy results. We treated the right side of the face with topical ketoconazole and the left side with topical corticosteroid. The pustules and erythema disappeared on the left side within about two days, although there were minimal effects on the right side (Figure 4). On the 10th day after birth, the patient’s blood test results showed an elevated white blood cell count (12,400/μL; reference range: 3400–9400/μL) and elevated eosinophil count (1240/μL; reference range: 0–658/μL), and pustules with erythema were found on the seborrheic areas of the trunk, such as the axillae and inguinal regions. About one month after birth, no new pustules or erythema were seen at the sites where topical corticosteroid had been applied, although new eruptions were still observed on the trunk.

## 3. Discussion

To diagnose noninfectious pustular diseases, infectious pustular diseases must be ruled out. Infectious pustular diseases include bullous impetigo, congenital cutaneous candidiasis, herpes simplex virus infection, varicella, etc. [1]. These diseases are differentiated based on a combination of systemic symptoms, bacterial and fungal cultures, direct microscopy, Tzanck test, and PCR analyses. 

ETN is a transient physiological rash found in about 40% of newborn infants [2]. Such rashes typically develop within 72 h after birth but are rarely seen at the time of delivery [3,4]. ETN is characterized by erythema and papules or pustules, 1–3 mm in diameter, on the face, trunk, and limbs. Since it does not affect the palms of the hands or soles of the feet, it is thought that an immune response to microbial colonies in hair follicles is an important etiologic factor [5]. The individual lesions commonly disappear within a few hours, without pigmentation [2,6]. ETN typically resolves within a week and is not accompanied by systemic symptoms; therefore, no special treatments are required.

TNPM is common in black neonates, characterized by flaccid 1–3 mm pustules on the chin, neck, trunk, and thighs beginning at birth [7]. These pustules quickly transition into pigmented macules, which usually fade within a month [6,8]. No specific therapies for TNPM are required as it resolves spontaneously, and no systemic symptoms are associated with the lesions [6].

Although a previous report proposed that TNPM and ETN may represent different points on the spectrum of the same disease [9], the pathogenic mechanisms underlying TNPM remain unclear.

EPF in infancy is a rare disease first reported by Lucky et al. in 1984 [10]. EPF is a disease that causes pruritic erythema, blisters, and pustules centered on hair follicles without systemic symptoms. The predominant site of EPF in infancy is the scalp, although seborrheic areas of the face, trunk, and limbs may also be affected. The skin manifestations of EPF are characterized by pustules measuring 1–3 mm accompanied by erythema, leading to the formation of plaques and pigmented spots. Unlike adult EPF, the lesions of EPF in infancy exhibit secondary crusting [6,11]. These eruptions follow a cyclical course from three months to five years [11]. The best way to quickly diagnose EPF without assessment of the clinical course is to perform a Tzanck smear of a pustule or skin biopsy. The pustules in EPF contain large numbers of eosinophils and are accompanied by dermal infiltration of eosinophil-dominated inflammatory cells around the hair follicles. If the lesions do not show folliculitis on biopsy, the condition is preferentially referred to as neonatal eosinophilic pustulosis (NEP) [12]. As EPF and NEP present with similar symptoms and a similar clinical course, it may not be necessary to distinguish between EPF and NEP. Topical corticosteroids were reported previously to show beneficial effects in both EPF and NEP [12]. 

Our patient presented with many pustules but relatively minimal crusting at birth compared to typical infant cases of EPF. This may be because our case was bathed in amniotic fluid without exposure to air at the onset of EPF. However, one week after birth, our case exhibited typical crusting pustules. According to the clinical course over one month, the negative results of pathogen tests, the findings of the skin biopsy, and the response to treatment, we could differentiate EPF from the other infectious and noninfectious pustular diseases described above.

Although the cause of EPF in infancy has not been clarified, the increased number of eosinophils in the peripheral blood during attacks is suggestive of an immunological pathomechanism [10]. A clinical study of human polyomavirus 6 as a potential etiological agent for EPF in adults suggested a hypothetical association between persistent antigenic stimuli from infectious agents and tissue eosinophilia through activation of T helper 2 cells [13].

EPF develops within 24 h after birth in about 25% of affected infants under one year of age; however, the symptoms are rarely seen at birth [10,11,14]. To our knowledge, only two cases of EPF since birth have been reported to date [10,14]. Both cases exhibited the same clinical presentation and course as ordinary EPF in infancy; however, these previous cases were not accompanied by any maternal medical history or drug treatments. It has not been elucidated whether maternal immune disorders and oral medications influence the pathogenesis of EPF. The mother in our case had Graves’ disease and was taking propylthiouracil and aspirin. In our case, the eruption worsened with an increase in blood eosinophil count, which may have been related to the mother’s oral medication. Several adult cases of drug-induced EPF have been reported, leading to increased blood eosinophils [15,16]. In addition, a previous study reported that patients with Graves’ disease have a genetic predisposition to Th2 predominance [17]. It is well known that patients suffering from Th2-dominant diseases often show increased levels of peripheral eosinophils. Therefore, the mother’s medical history may have affected the pathogenesis of our patient’s EPF. Further case studies are required to shed light on the involvement of these conditions in the onset of the disease.

## Data Availability

The data presented in this study are available on request from the corresponding author. The data are not publicly available due to privacy restrictions.
